# Supplementary figures and images for: Organelle proteomic profiling reveals lysosomal heterogeneity in association with longevity
Source: eLife. 2024 Jan 19;13:e85214. doi: 10.7554/eLife.85214 (PMC10876212; doi:10.7554/eLife.85214)

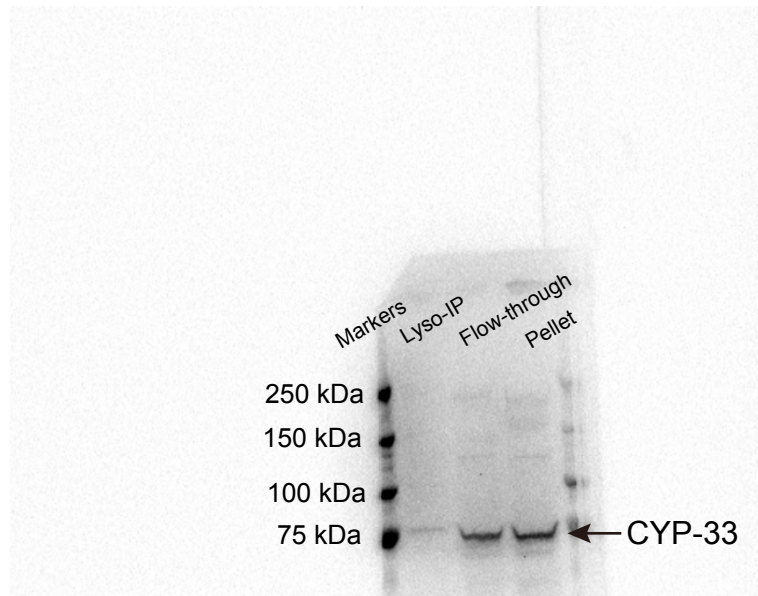

Supplement: Figure 1—source data 1. [file elife-85214-fig1-data1.zip › 85214Figures1SourceData1/CYP-33_labelled.pdf]

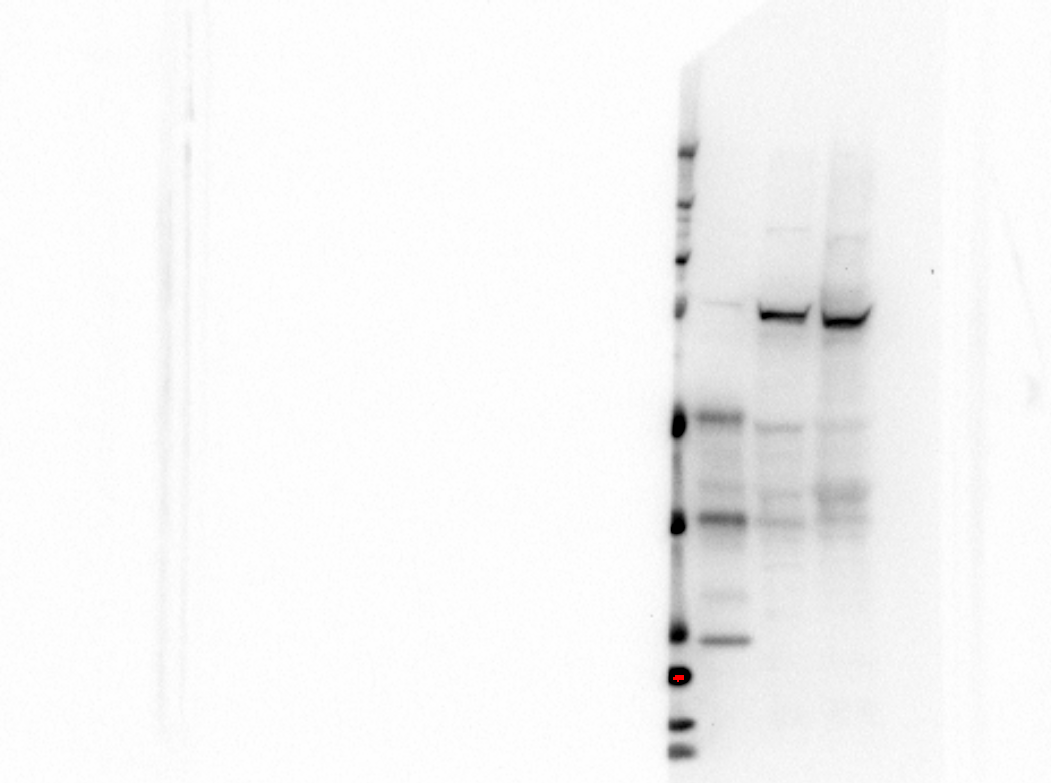

Supplement: Figure 1—source data 1. [file elife-85214-fig1-data1.zip › 85214Figures1SourceData1/LMP-1.tif]

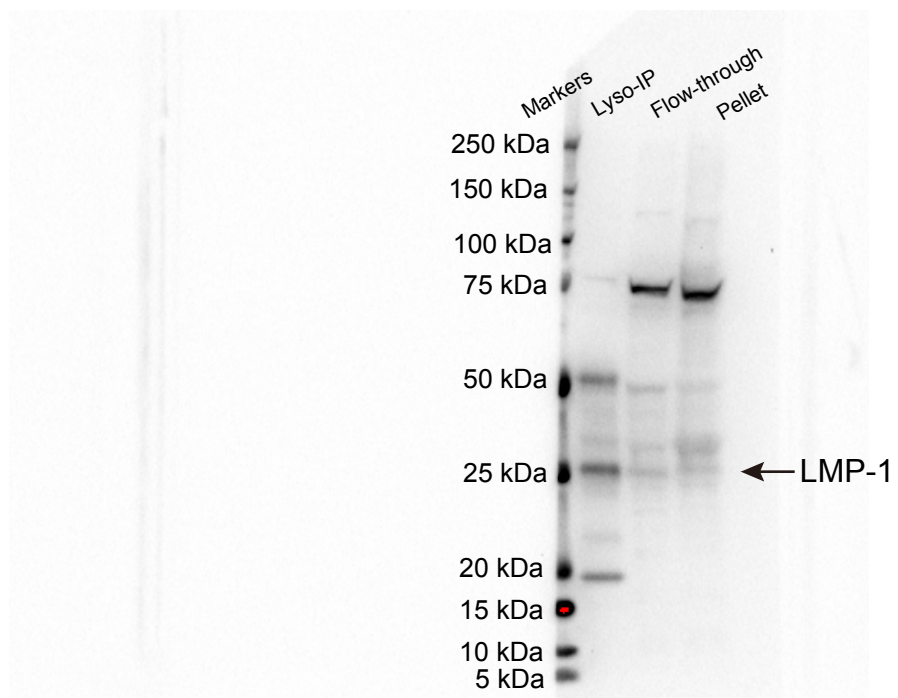

Supplement: Figure 1—source data 1. [file elife-85214-fig1-data1.zip › 85214Figures1SourceData1/LMP-1_labelled.pdf]

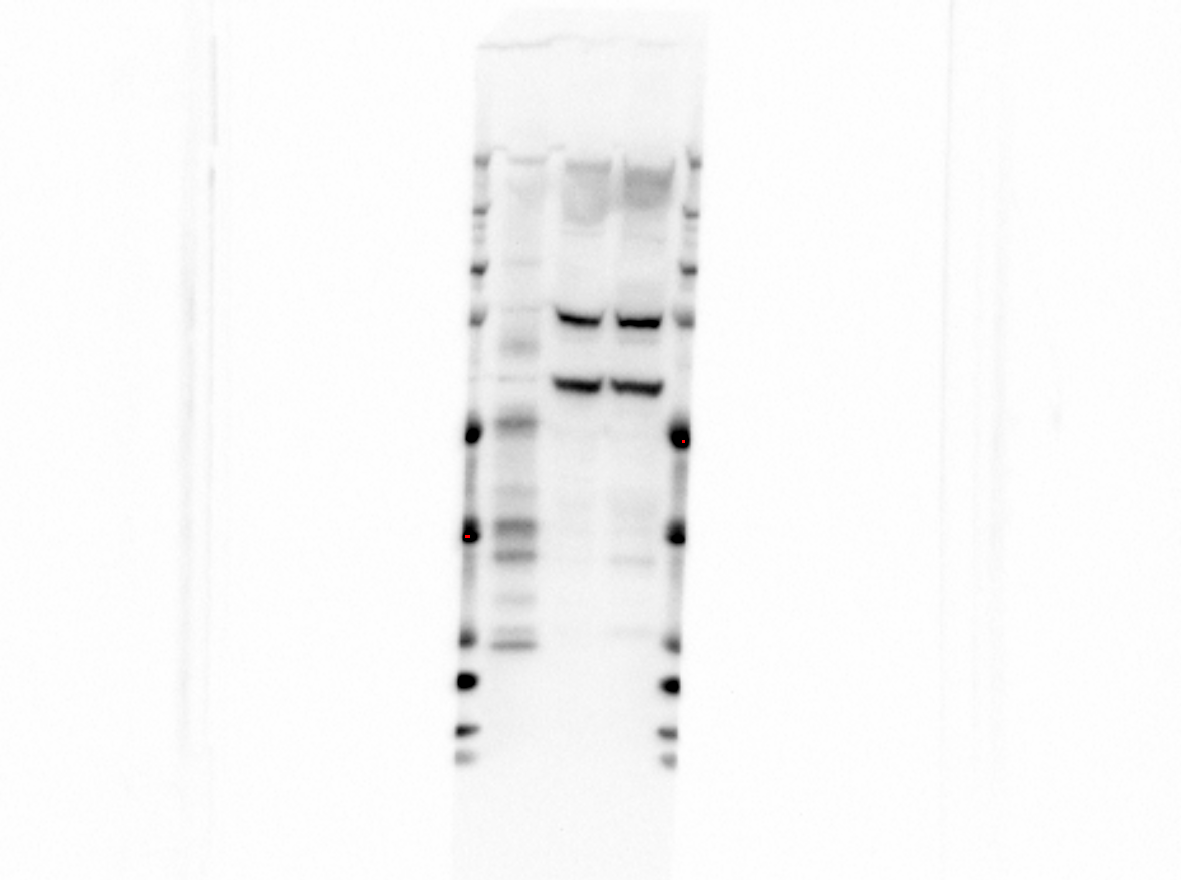

Supplement: Figure 1—source data 1. [file elife-85214-fig1-data1.zip › 85214Figures1SourceData1/HSP-60.tif]

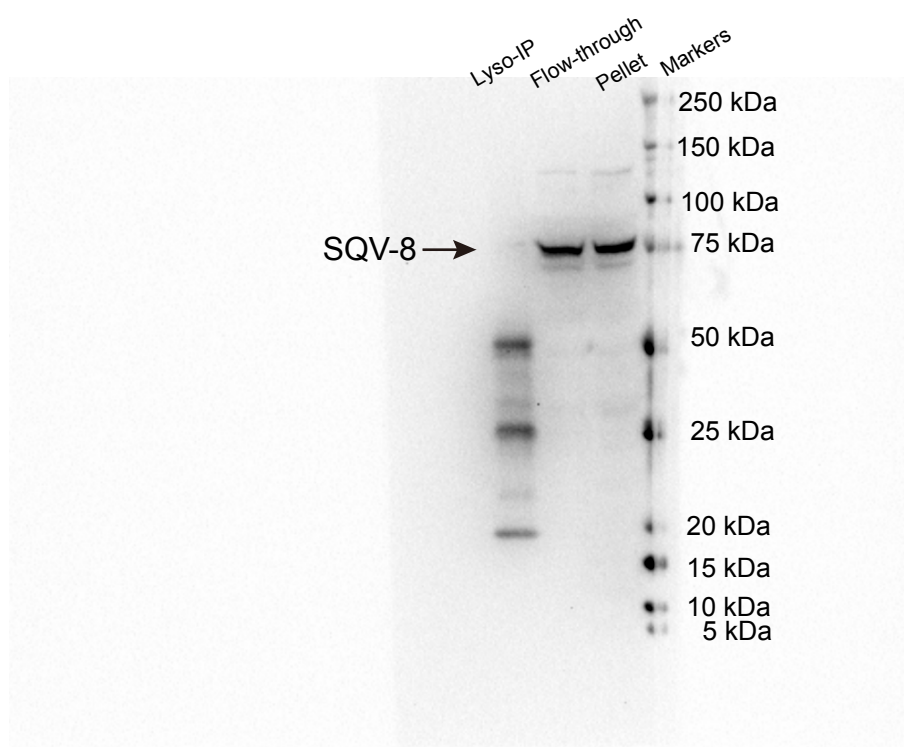

Supplement: Figure 1—source data 1. [file elife-85214-fig1-data1.zip › 85214Figures1SourceData1/SQV-8_labelled.pdf]

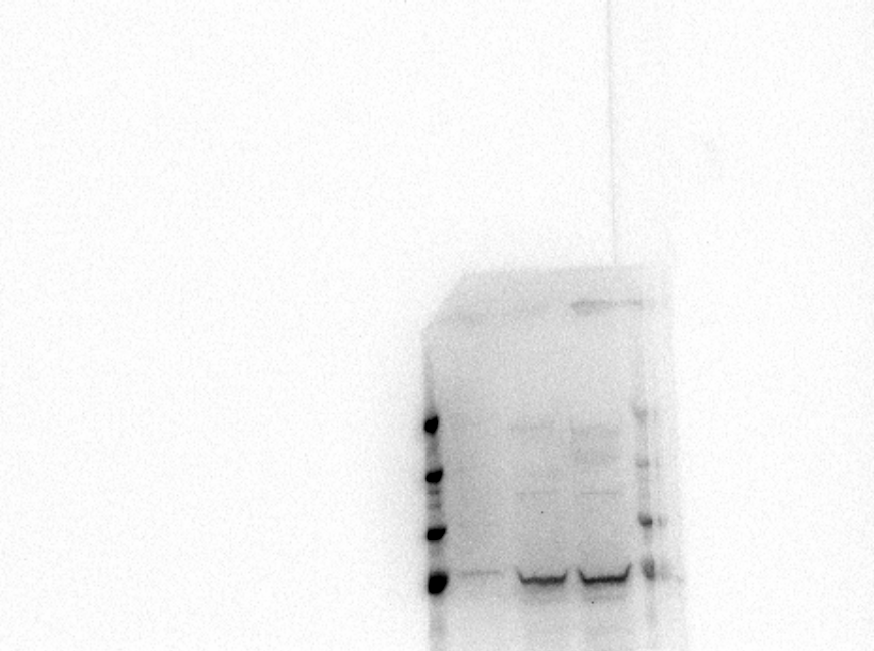

Supplement: Figure 1—source data 1. [file elife-85214-fig1-data1.zip › 85214Figures1SourceData1/CYP-33.tif]

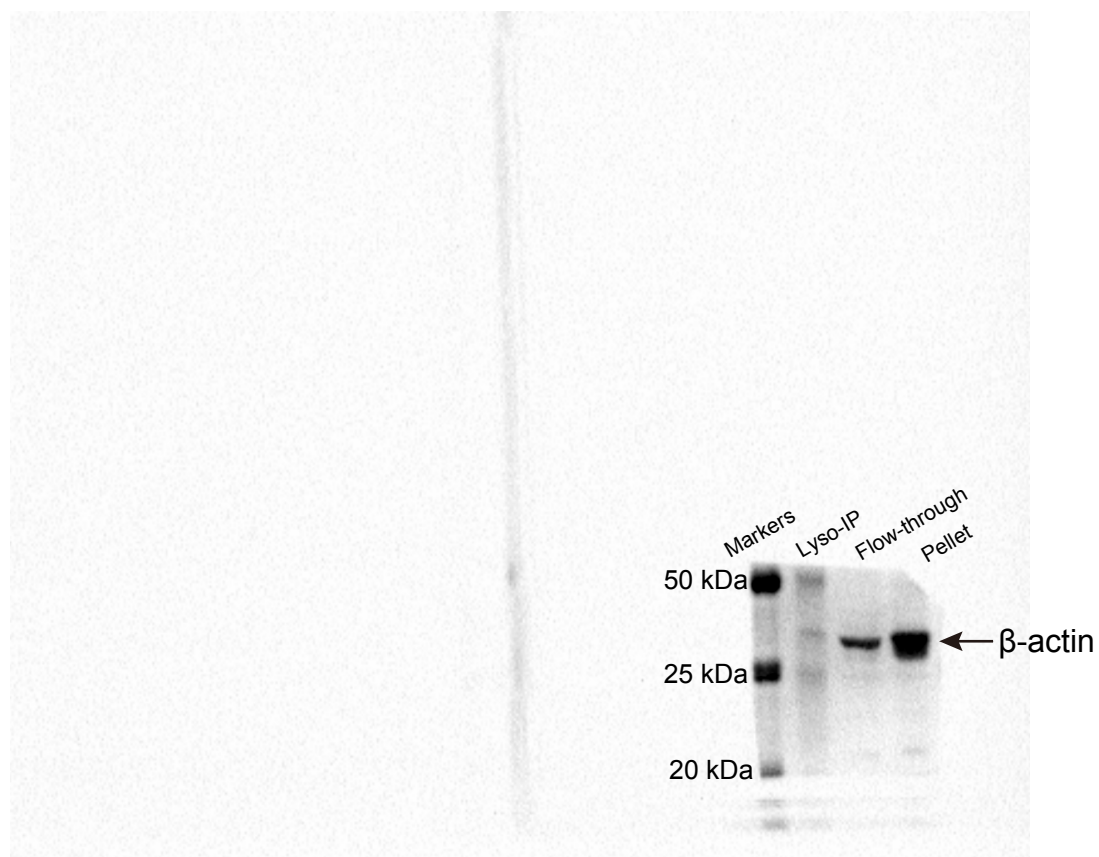

Supplement: Figure 1—source data 1. [file elife-85214-fig1-data1.zip › 85214Figures1SourceData1/╬▓-actin_labelled.pdf]

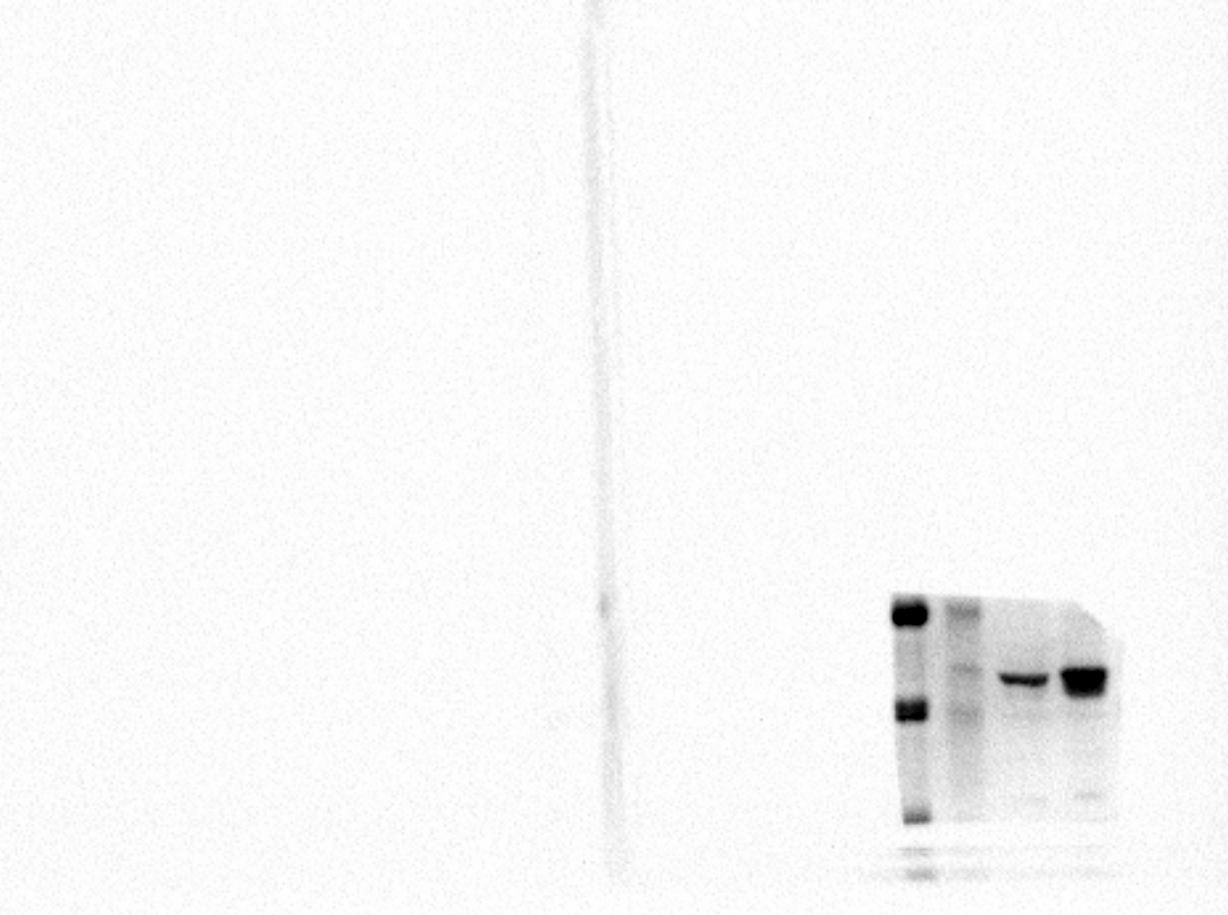

Supplement: Figure 1—source data 1. [file elife-85214-fig1-data1.zip › 85214Figures1SourceData1/╬▓-actin.tif]

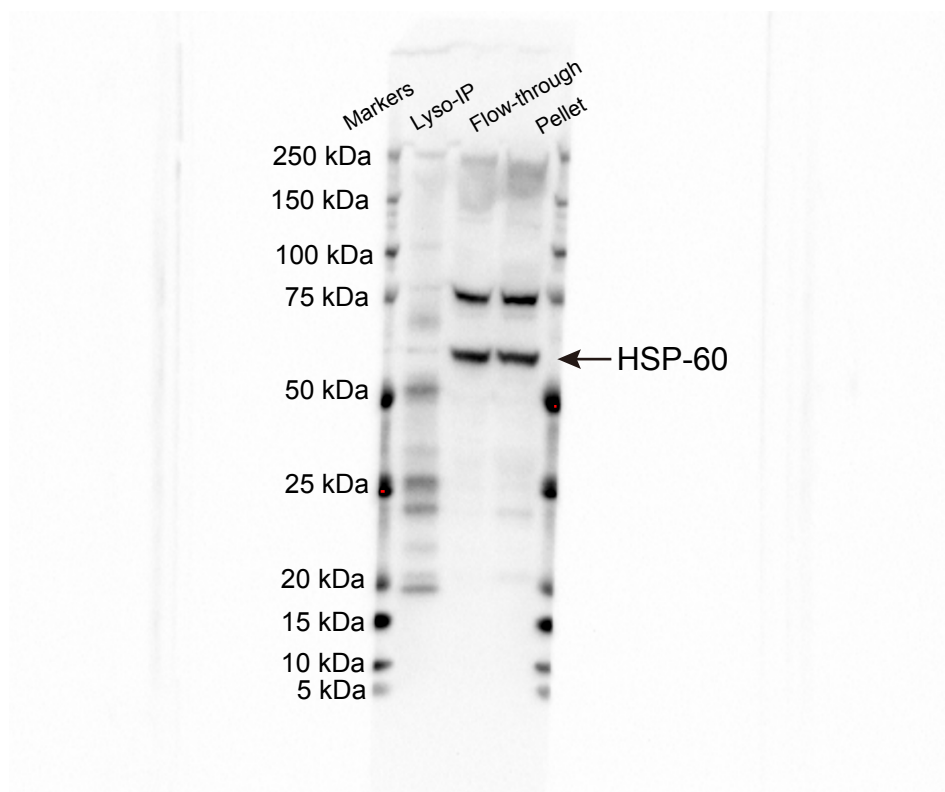

Supplement: Figure 1—source data 1. [file elife-85214-fig1-data1.zip › 85214Figures1SourceData1/HSP-60_labelled.pdf]

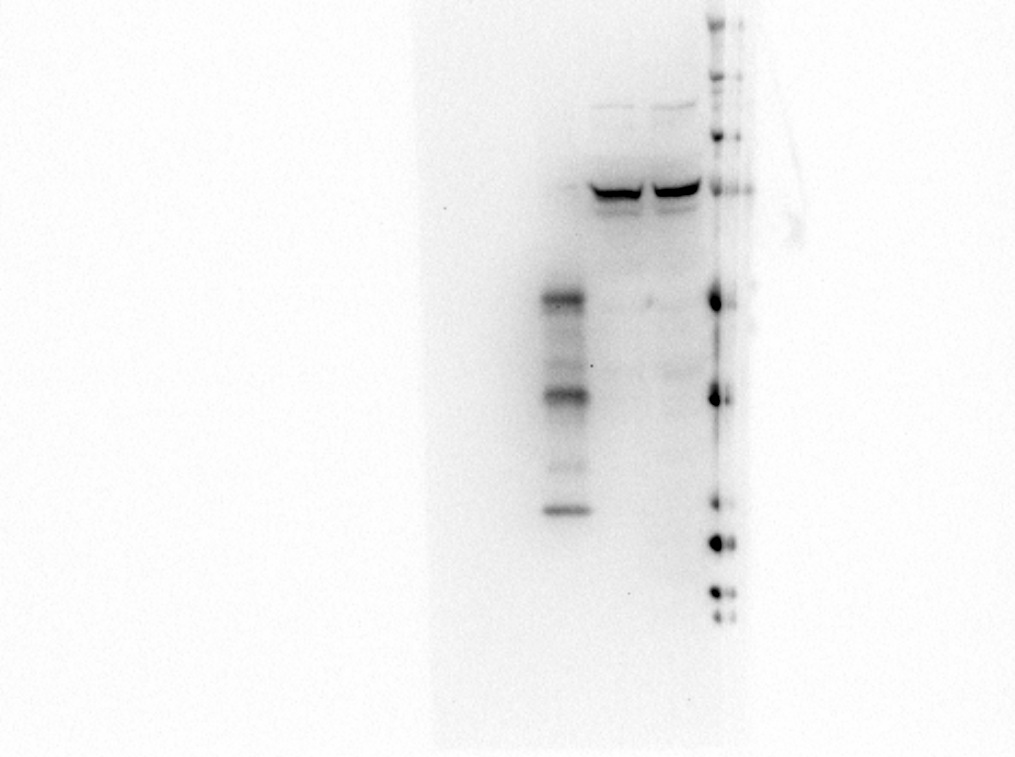

Supplement: Figure 1—source data 1. [file elife-85214-fig1-data1.zip › 85214Figures1SourceData1/SQV-8.tif]
